# Worldwide productivity and research trend of publications concerning SIAD: a bibliometric study

**DOI:** 10.3389/fendo.2024.1297164

**Published:** 2024-03-05

**Authors:** Xiaodan Wei, Weiyuan Pan, Shaocong Lun, Yuexuan Wu, Yushi Chen, Taoshan Feng, Meilian Liu, Xiaoming Chen

**Affiliations:** ^1^ Department of Hospice, Affiliated Hospital of Guangdong Medical University, Zhanjiang, Guangdong, China; ^2^ First College of Clinical Medicine, Guangdong Medical University, Zhanjiang, Guangdong, China; ^3^ Department of Pulmonary Oncology, Affiliated Hospital of Guangdong Medical University, Zhanjiang, Guangdong, China; ^4^ Department of Endocrinology, Affiliated Hospital of Guangdong Medical University, Zhanjiang, Guangdong, China; ^5^ Faculty of Chinese Medicine, Macau University of Science and Technology, Avenida Wai Long, Taipa, Macau, China

**Keywords:** SIAD, bibliometric analysis, development, VOSviewer, CiteSpace

## Abstract

**Background:**

Syndrome of inappropriate antidiuretic(SIAD) occurs secondary to various diseases, which is characterised by hypotonic hyponatremia and impaired urinary diluting capacity. Research on SIAD in both domestic and international contexts has a long history. This study objectively and comprehensively analyses the research trends, hotspots and development of SIAD research of the past 20 years using the method of bibliometric analysis.

**Methods:**

The 2003–2022 data in the Web of Science Core Collection database were searched. The Bibliometrix software package, VOSviewer and CiteSpace were used to mine, extract and visualise the retrieved literature, and the generated maps were used in analysing the main topics and trends in the field of SIAD research.

**Results:**

A total of 1215 articles published in 623 journals were included in the analysis, with a total of 18,886 citations. Results showed that the research output on SIAD has continuously increased in the past 20 years, and the United States had the highest number of publications and citations. Keywords with the highest burst strength in recent years were the most mentioned keywords, in addition to the search terms ‘hyponatremia’, ‘covid-19’, and ‘mortality’. Thus, the relationship among SIAD, covid-19 and mortality may become research frontiers and trends. Fifteen milestone articles were identified through co-citation analysis, which mainly focused on the pathophysiology and treatment of SIAD.

**Conclusion:**

Based on bibliometric analysis and knowledge mapping, this study summarises development trends in the field of SIAD research, providing references for current and future research into SIAD.

## 1 Introduction

Syndrome of inappropriate antidiuretic (SIAD), also known as syndrome of inappropriate antidiuresis, is the most common cause of hypotonic hyponatremia. SIAD results from an excess of AVP activity beyond osmotic requirements (1). Its main features include water retention, hypo-osmolar hyponatremia, increased urinary sodium and elevated urine osmolality. The symptoms of SIAD depend on the etiology, the rate of disease progression, and the severity and duration of hyponatremia (2). Acute hyponatremia can lead to serious complications such as seizures, reflex inhibition, coma, and even death (1, 3). On the other hand, chronic hyponatremia include nonspecific symptoms like nausea, vomiting, dizziness, weakness, and headache. It may be not severe or life-threatening, but is associated with cognitive impairment, osteoporosis, falls, fractures (4–6).The main causes of SIAD include tumours, drugs, neurological disorders, pulmonary diseases, and other factors (2). Hyponatremia caused by SIAD can be easily overlooked in clinical settings.

Medical bibliometrics refers to the quantitative or semiquantitative analysis of literature, considering quantity, quality and relationships among authors (7). Numerous reports on bibliometrics have been published in various fields. However, despite that some articles related to SIAD have been published, they lack sufficient quantitative analysis, and no systematic evaluation of the relevant literature has been conducted yet. Therefore, this study aims to objectively and comprehensively analyse research trends, hot topics and development trajectories in SIAD research. Using medical bibliometrics analysis, we summarised the current status of SIAD research and identify potential research directions.

## 2 Material and methods

### 2.1 Data source and search

This study data were obtained from the Web of Science Core Collection database. Literature retrieval and downloading were conducted on 26 June 2023 by advanced search tools. The key topic for retrieval was TS = (‘syndrome of inappropriate antidiuretic hormone secretion’ [topic] or ‘syndrome of inappropriate antidiuresis’ [topic] or SIADH [topic] or SIAD [topic]). The retrieval procedure was set to include only articles published in English from 2003 to 2022. A total of 1215 articles were retrieved, exported and stored. These processes were independently completed and verified by two authors, who discussed any discrepancies until a consensus was reached.

### 2.2 Data analysis and visualisation

The study applied bibliometric methods to analyse research trends, hot topics and development patterns in SIAD research. R version 4.2.3, VOSviewer version 1.6.19, CiteSpace version 5.7.r5 and Pajek scientific were used in bibliometric analysis and visualisation of the collaborative relationships among countries, institutions and authors. Besides, these tools were used in analysing the co-occurrence and clustering of keywords and the co-citation analysis of the literature. Additionally, the network tool ‘MapInSeconds’ by Eugene Chen from Black Horse Analysis Company (mapinseconds.com) was used in creating a geographical visualisation of publication outputs by countries.

## 3 Results

### 3.1 Annual publication output

**Figure 1:**
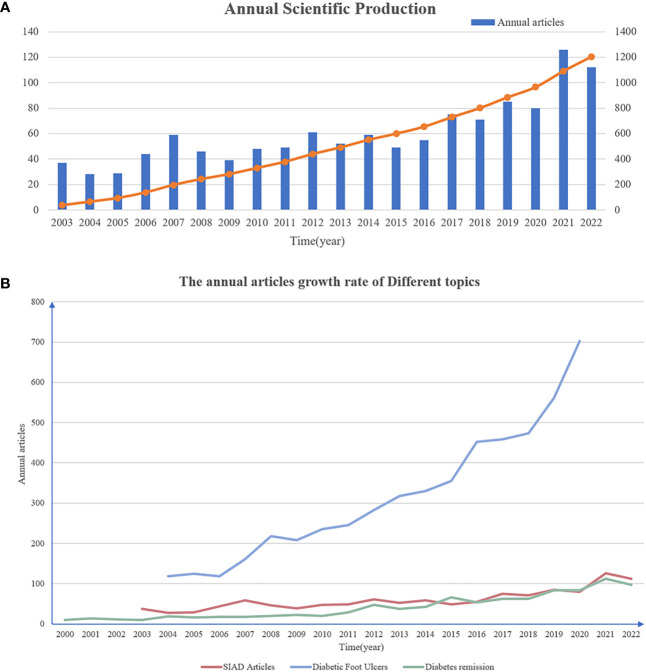
Production of annual articles. **(A)** The annual scientific production in SIAD research from 2003 to 2022. The blue column represents the annual articles and the orange curve represents the cumulative articles. **(B)** The comparison of publication growth rate between SIAD with diabetic foot ulcers and diabetes remission. The red curve represents the annual articles in SIAD research. The light blue curve represents the annual articles in diabetic foot ulcers research from 2004 to 2020 and the light green curve represents the annual articles in diabetes remission research from 2000 to 2022.

According to the data retrieved from WoSCC, 1215 articles related to SIAD were identified from January 2003 to December 2022. The research trends were roughly divided into three stages. The first stage spanned from 2003 to 2011, during which 379 articles were published for over 9 years, and showed a steady and moderate growth rate. In the second stage, although the publication output slightly fluctuated, the overall trend continued to show growth. The third stage, from 2019 to 2022, exhibited the highest growth rate in terms of publication output (**Figure 1A**). The number of articles published reached its peak in 2021, which had 126 articles that accounted for approximately 10.0% of the total. **Figure 1B** shows the comparison of publication growth rate between SIAD with other common or hot topic. The growth rate in SIAD publication was slower than that in diabetic foot ulcers publication but it was similar to diabetes remission publication (8, 9). In summary, over the past two decades, research related to SIAD has shown an overall increasing trend, indicating steady development in this area.

### 3.2 Analysis of countries/regions

**Figure 2:**
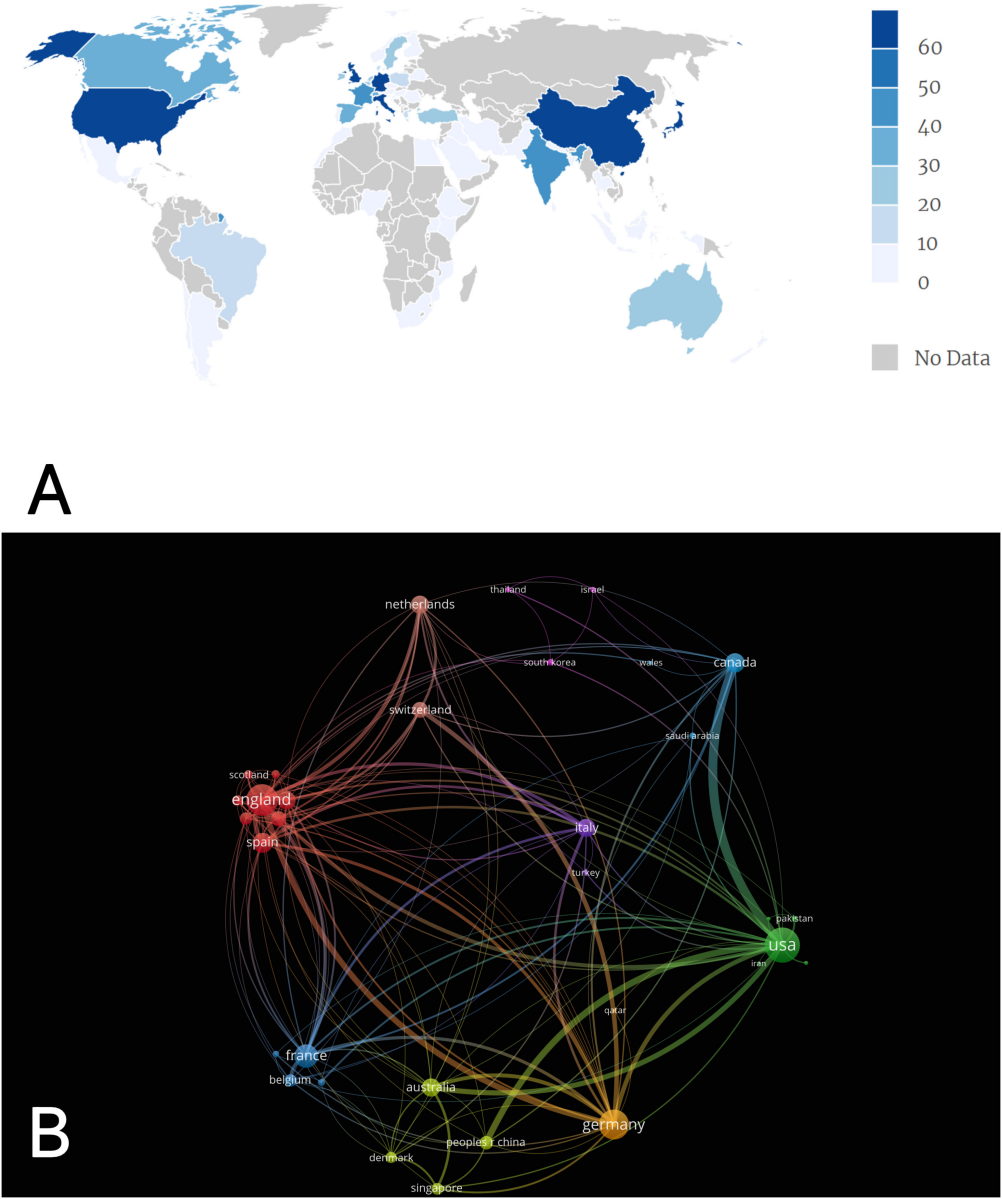
Analysis of countries engaged in SIAD. **(A)** Geographical distribution of article publications related to SIAD. The depth of colour matched with the number of articles. **(B)** Cooperation of countries or regions on SIAD research from 2003 to 2022.Each node represents a country or region. The link size refers to the cooperation intensity. The size of the node is proportional to the total link strength.

The 1215 articles included in this study were authored by researchers from 63 countries or regions participating in SIAD research according to the Bibliometrix results. **Figure 2A** shows the geographical visualisation of the publication output by countries. Dark colours represent high publication output, and grey areas indicate countries or regions without relevant publications. The top 10 countries/regions in SIAD field were shown in **Table 1**. The United States not only had the highest publication output but also had the highest total and average citation counts, indicating significant contributions to the advancement and development of SIAD research. Japan ranked second despite having a much lower publication output than the United States, accounting for approximately 10%. China published 74 articles, representing 6.1%, and ranked third. European countries, such as Italy, Germany and the United Kingdom, had relatively comparable publication outputs, suggesting similar contributions to this field.

**Table 1:** The top 10 countries/regions in SIAD field.

Country	Articles	Article/TP	Total Citations	Average Article Citations
USA	264	0.217	6766	25.60
JAPAN	121	0.10	862	7.10
CHINA	74	0.061	482	6.50
ITALY	68	0.056	687	10.10
GERMANY	66	0.054	1201	18.20
UNITED KINGDOM	64	0.053	1294	20.20
INDIA	43	0.035	234	5.40
FRANCE	41	0.034	501	12.20

TP:The total number of articles.

By using VOSviewer, we constructed a co-occurrence visualisation of country or regional collaboration (**Figure 2B**). Each node represents a country or region, and the link between two nodes represent a collaborative relationship between two countries. Node size represents total link strength. A close collaborative relationships were found among various countries or regions in the field of SIAD research. Notably, the node representing the United States is the largest, indicating that its total link strength is higher than that of other countries, signifying close collaboration between the United States and other countries or regions. Additionally, China’s main collaborating countries are the United States and Canada. However, the total link strength is much lower than that of the United States and Canada, indicating that China needs to engage in more extensive collaboration to guide scientific research activities and optimise collaboration structures and thereby promote the development and advancement of SIAD research.

### 3.3 Analysis of authors and institutions collaboration network

A total of 33 authors published five or more articles about SIAD. **Table 2** presents the top 10 authors based on their publication output and total citation counts. Christ-crain M (n = 16) and Decaux G (n = 16) had the highest publication outputs, followed by Skov J (n = 14). Verbalis JG published 12 articles and shared the 8th position with Sherlock M, but Verbalis JG’s articles had the highest total citation count, reaching 520 citations. This indicates that Verbalis JG’s research in the SIAD field has the most significant impact.

**Table 2:** The top 10 productive authors and co-cited authors in SIAD research.

Rank	Author	Documents	Rank	Co-authors	Citations
1	Christ-crain M	16	1	Verbalis JG	520
1	Decaux G	16	2	Decaux G	328
3	Skov J	14	3	Schrier RW	219
4	Calissendorff J	13	4	Sterns RH	204
4	Falhammar H	13	5	Adrogué HJ	184
4	Lindh JD	13	6	Ellison DH	174
4	Mannheimer B	13	7	Hoorn EJ	155
8	Sherlock M	12	8	Spasovski G	152
8	Verbalis JG	12	9	Robertson GL	148
10	Cuesta M	9	10	Maesaka JK	127

**Figure 3:**
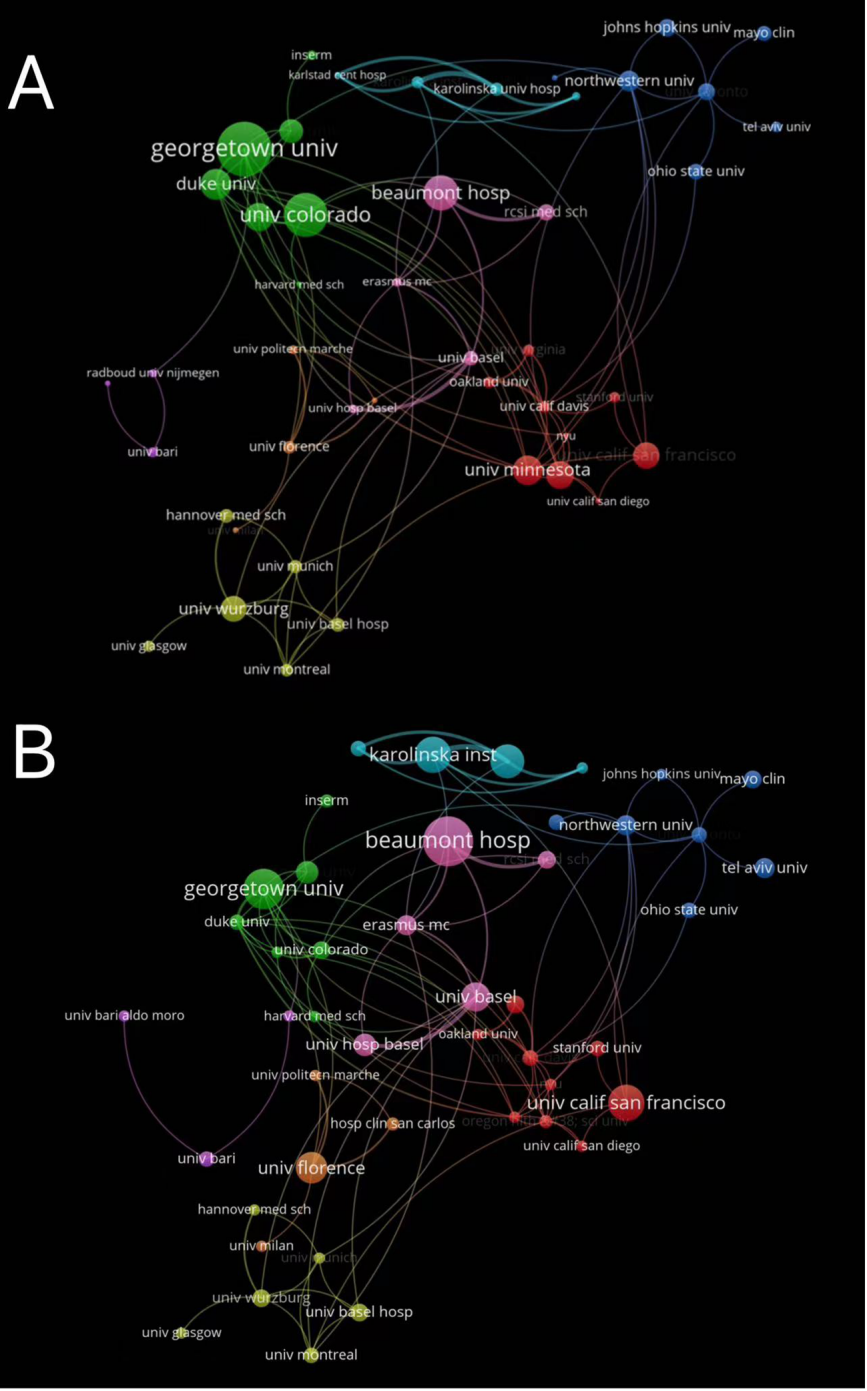
Analysis of authors and institutions engaged in SIAD. **(A)** The visualization map of collaborations between institutions. The lines between nodes represent cooperation between institutions. Each node represents one institution. The size of the node is proportional to the number of documents. **(B)** The visualization map of collaborations between institutions. The size of the node is proportional to the number of citations.

As shown in **Figure 3A**, the top two institutions or affiliated institutions in terms of publication output were Beaumont Hospital (n = 22) and Georgetown University (n = 18). Karolinska Institution (n = 16) and University of California, San Francisco (n = 16) are tied for the third position. In terms of citation count (citation), Georgetown University (1959 citations) ranked first, followed by University of Colorado (1339 citations), and Beaumont Hospital (917 citations) ranked last (**Figure 3B**). The institutions with close collaborations were Karolinska Institution and Karolinska University Hospital, which had total link strength of 27 and 26. Both institutions belong to a same organization, which may account for their close cooperation. By contrast, collaborations among other institutions were scattered, indicating lack of strong collaboration among institutions or affiliated hospitals conducting research into SIAD.

### 3.4 Analysis of journals

According to Bibliometrix analysis, the 1215 articles included in this study were published in 623 journals. The journal with the highest publication output was ‘INTERNAL MEDICINE’ (30/1215, 2.4%), whereas the most cited journal was the ‘AMERICAN JOURNAL OF MEDICINE’ with 1207 citations. The H-index, also known as the H-factor or H-index, is a composite score used in assessing the importance, significance and widespread impact of a scientist’s cumulative research contributions (10). Based on the H-index results, **Table 3** presents the top 10 journals with the most published articles. The journal with the highest H-index score was ‘JOURNAL OF CLINICAL ENDOCRINOLOGY & METABOLISM’ from the United States (11), followed by the ‘AMERICAN JOURNAL OF MEDICINE’ also from the United States (10). According to Journal Citation Reports 2022 standards, seven journals from **Table 3** were in the Q1 zone. The journals with the highest impact factors were ‘JOURNAL OF THE AMERICAN SOCIETY OF NEPHROLOGY’ and ‘AMERICAN JOURNAL OF KIDNEY DISEASES’, indicating that most of the SIAD-related articles were published in authoritative journals.

**Table 3:** The top 10 productive journals of SIAD research with the highest score of h_index.

Journal	h_index	TC	NP	PY_start	2022JCR	IF
JOURNAL OF CLINICAL ENDOCRINOLOGY \& METABOLISM	11	589	17	2003	Q1	5.8
AMERICAN JOURNAL OF MEDICINE	10	1207	10	2006	Q1	5.9
AMERICAN JOURNAL OF KIDNEY DISEASES	9	277	14	2004	Q1	13.2
CLINICAL ENDOCRINOLOGY	9	394	10	2006	Q3	3.2
EUROPEAN JOURNAL OF ENDOCRINOLOGY	9	560	11	2003	Q1	5.8
BEST PRACTICE & RESEARCH CLINICAL ENDOCRINOLOGY & METABOLISM	7	454	8	2003	Q1	9.8
ENDOCRINE	7	104	11	2009	Q3	3.7
EUROPEAN JOURNAL OF INTERNAL MEDICINE	7	187	9	2007	Q1	8
INTERNAL MEDICINE	7	173	30	2003	Q4	1.2
JOURNAL OF THE AMERICAN SOCIETY OF NEPHROLOGY	7	308	7	2007	Q1	13.6

TC, Total Citation; NP, The number of publication; PY_start, The year for the first publication.

**Figure 4:**
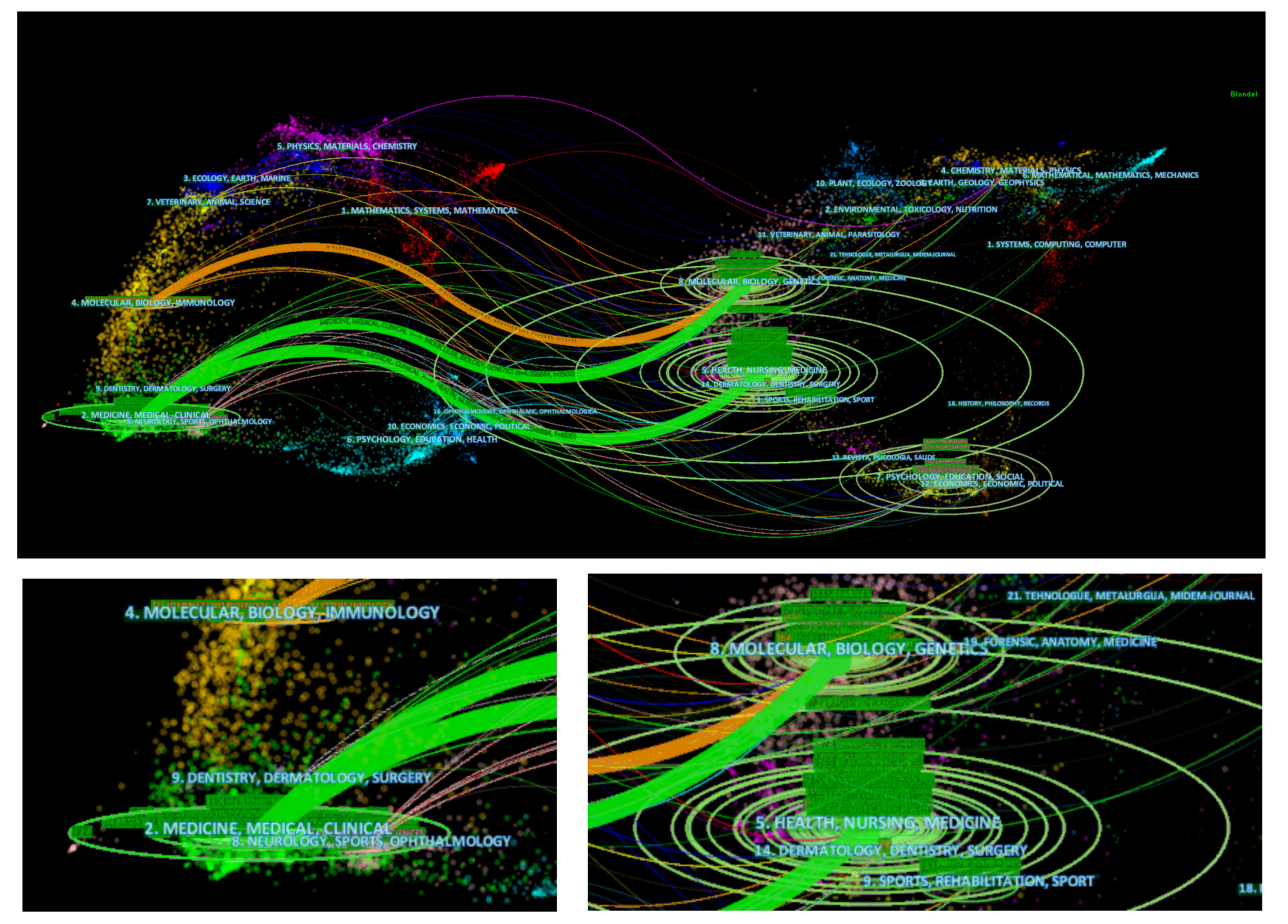
The dual map of journals published on SIAD. Citing journals are on the right, cited journal is on the left, and lines represent the citation relationship.

The dual-map overlay of the journals about SIAD were presented in CiteSpace (**Figure 4**) for the visualisation of subjects related to the journals, as well as the relationship between the source journals on the left and the target journals on the right. The colourful links represented the citation relationship between journals. The three main citation pathways were identified, comprising two green paths and one orange path. The green paths indicated that studies involve journals related to Molecular/Biology/Genetics and Health/Nursing/Medicine were often cited by Medicine/Medical/Clinical (**Figure 4**). The orange path showed that research involve journals related to Molecular/Biology/Genetics were frequently cited by Molecular/Biology/Immunology journals.

### 3.5 Analysis of references

**Figure 5:**
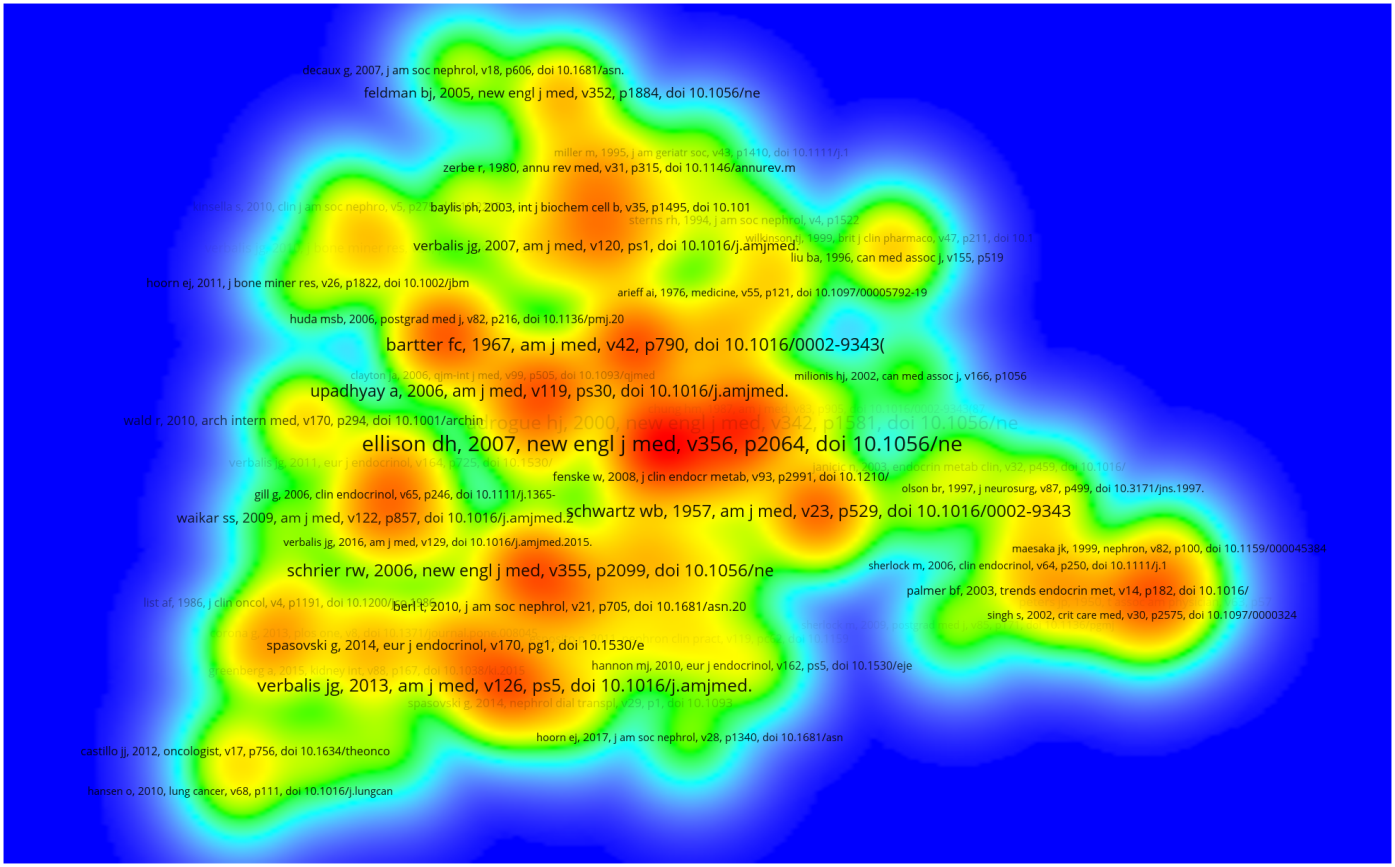
The density visualization map of the top 73 co-cited references from 2003 to 2022. Red references represent the highest frequency.

Two or more references simultaneously cited by one or more articles are known as a co-citation relationship. The more frequently a literature is cited, the more significant it is in a particular field. A total of 18,886 references were extracted from 1215 articles. Owing to the large number of cited references, the minimum number of citations was set at 25, and only 73 cited references met the conditions and thus selected for co-citation analysis (**Figure 5**). The colour intensity of each reference indicated the number of co-citations. The red references represented the highest frequency. Among the top 10 most frequently co-cited references in **Table 4** (3, 4, 11–18), six references were cited over 100 times. The top two references were published in NEW ENGLAND JOURNAL OF MEDICINE. The former was a guideline on the diagnosis and treatment of SIAD and established by Ellison DH et al. in 2007 (11). The latter was a summary of diagnosis and treatment of hyponatremia and proposed by Adrogué, HJ, et al. in 2000 (2, 12).

**Table 4:** The top 10 co-cited references on SIAD research.

Rank	Cited References	Journals	Year	Citations
1	Clinical practice. The syndrome of inappropriate antidiuresis (11).	NEW ENGLAND JOURNAL OF MEDICINE	2007	172
2	Hyponatremia (12).	NEW ENGLAND JOURNAL OF MEDICINE	2000	136
3	The syndrome of inappropriate secretion of antidiuretic hormone (13).	AMERICAN JOURNAL OF MEDICINE	1967	123
4	Diagnosis, evaluation, and treatment of hyponatremia: expert panel recommendations (14).	AMERICAN JOURNAL OF MEDICINE	2013	115
5	Tolvaptan, a selective oral vasopressin V2-receptor antagonist, for hyponatremia (15).	NEW ENGLAND JOURNAL OF MEDICINE	2006	111
6	A syndrome of renal sodium loss and hyponatremia probably resulting from inappropriate secretion of antidiuretic hormone (16).	AMERICAN JOURNAL OF MEDICINE	1957	111
7	Incidence and prevalence of hyponatremia (17).	AMERICAN JOURNAL OF MEDICINE	2006	97
8	Mild chronic hyponatremia is associated with falls, unsteadiness, and attention deficits (4)	AMERICAN JOURNAL OF MEDICINE	2006	94
9	Clinical practice guideline on diagnosis and treatment of hyponatraemia (3).	EUROPEAN JOURNAL OF ENDOCRINOLOGY	2014	66
10	Mortality after hospitalization with mild, moderate, and severe hyponatremia (18).	AMERICAN JOURNAL OF MEDICINE	2009	66

**Figure 6:**
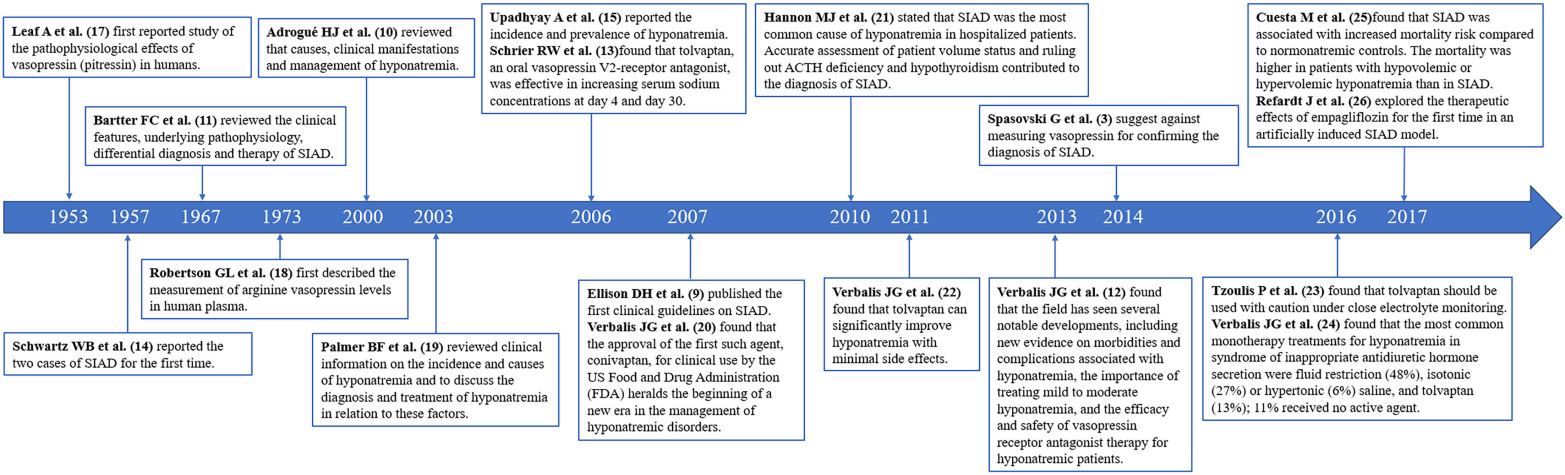
Timeline of part of landmark achievements in SIAD research.

Based on historiograph of R version and co-citation analysis, 18 landmark articles in SIAD field were identified (**Figure 6**) (3, 11–17, 19–28). These articles represent major nodes in SIAD research. The first case of SIAD was detected in 1957 (16) and the diagnostic criteria for SIAD were initially proposed in 1967 (13). It was not until 2007 that the first clinical guidelines on SIAD were published in the New England Journal of Medicine (11). In 2014, the European guidelines on hyponatremia highlighted the difficulty in measuring vasopressin concentration and, for this reason, did not recommend it as one of the diagnostic criteria for SIAD (3). After extensive research, fluid restriction remains the primary treatment for SIAD. The second line SIAD therapy include urea, tolvaptan, and SGLT2 inhibitors (1). The selection of these treatment methods still deserves attention and further study.

### 3.6 Analysis of keywords

**Figure 7:**
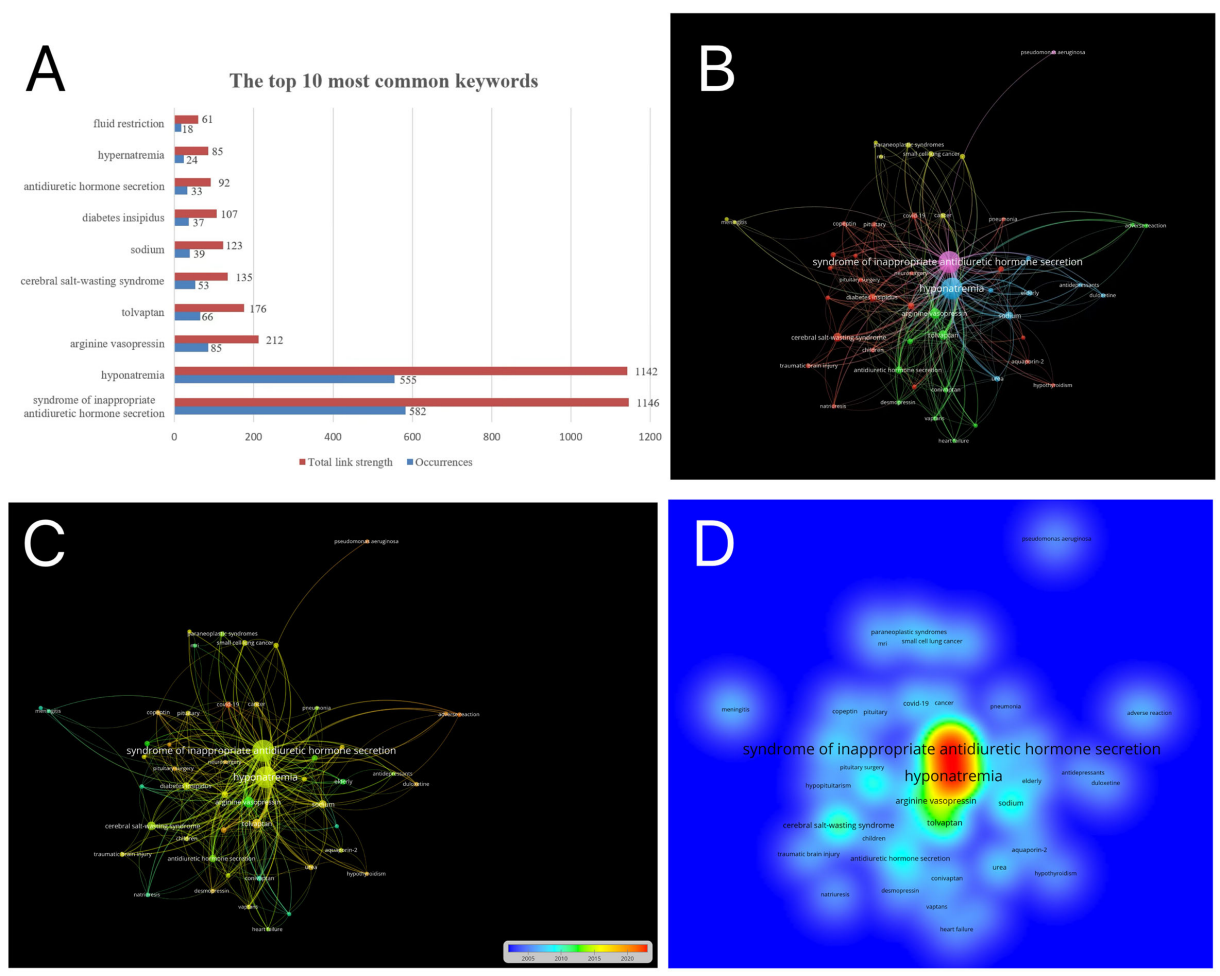
The bibliometric analysis of the keywords. **(A)** The top 10 most common keywords. **(B)** Co-occurrence analysis of keywords shown in different clusters in SIAD area. Different clusters are represented by different colours. Each node represents one keyword. The size of the node is proportional to the frequency of keyword. The line between two nodes represents that both keywords occurred in one article. **(C)** The visualization map of time when a keyword appeared. Keywords in blue appeared earlier than that in red. **(D)** The density visualization map of Keyword distribution according to the average frequency of occurrence. Red keywords represent the highest frequency.

Keywords represent main research topics. VOSviewer was used to count keywords extracted from the 1215 articles. Some similar keywords were merged, and meaningless terms were excluded. The top 10 high-frequency keywords are shown in **Figure 7A**. ‘SIADH’, ‘hyponatremia’, ‘arginine vasopressin’, ‘tolvaptan’ and ‘sodium’ had high frequencies, which indicated that the keyword are used and discussed frequently in SIAD research. Keyword co-occurrence refers to the frequency of two or more keywords appearing in the same literature (29). When the frequency of occurrence was set at 8, a total of 50 terms reached the threshold. A keyword co-occurrence map was constructed by VOSviewer (**Figure 7B**). Each node represented a keyword, and the size of the node was positively correlated with the frequency of occurrence. The lines represented the frequencies of the connected keywords, and thick lines indicated high co-occurrence frequencies. Different colours represented different cluster groups. As shown in **Figure 7B**, the extracted keywords were mainly classified into five clusters. Cluster 1 (red colour) was mainly involved differential diagnosis related to SIAD. Cluster 2 (yellow) was primarily related to the etiology of SIAD. Cluster 3 (green) was focused on the treatment and prognosis of SIAD. Although clusters 4 (pink) and 5 (blue) had fewer keywords, they included the two largest nodes: ‘SIADH’ and ‘hyponatremia’. The overlay visualisation based on the publication time of keywords is shown in **Figure 7C**, where different node colours represent the average year of appearance for each keyword. The adverse reaction and treatment of SIAD have attracted considerable interest, as well as the interrelations between SIAD and covid-19, in recent years. **Figure 7D** shows the density map of keywords measured by the colour intensity. The hotspots in this research field were often located in the red area.

**Figure 8:**
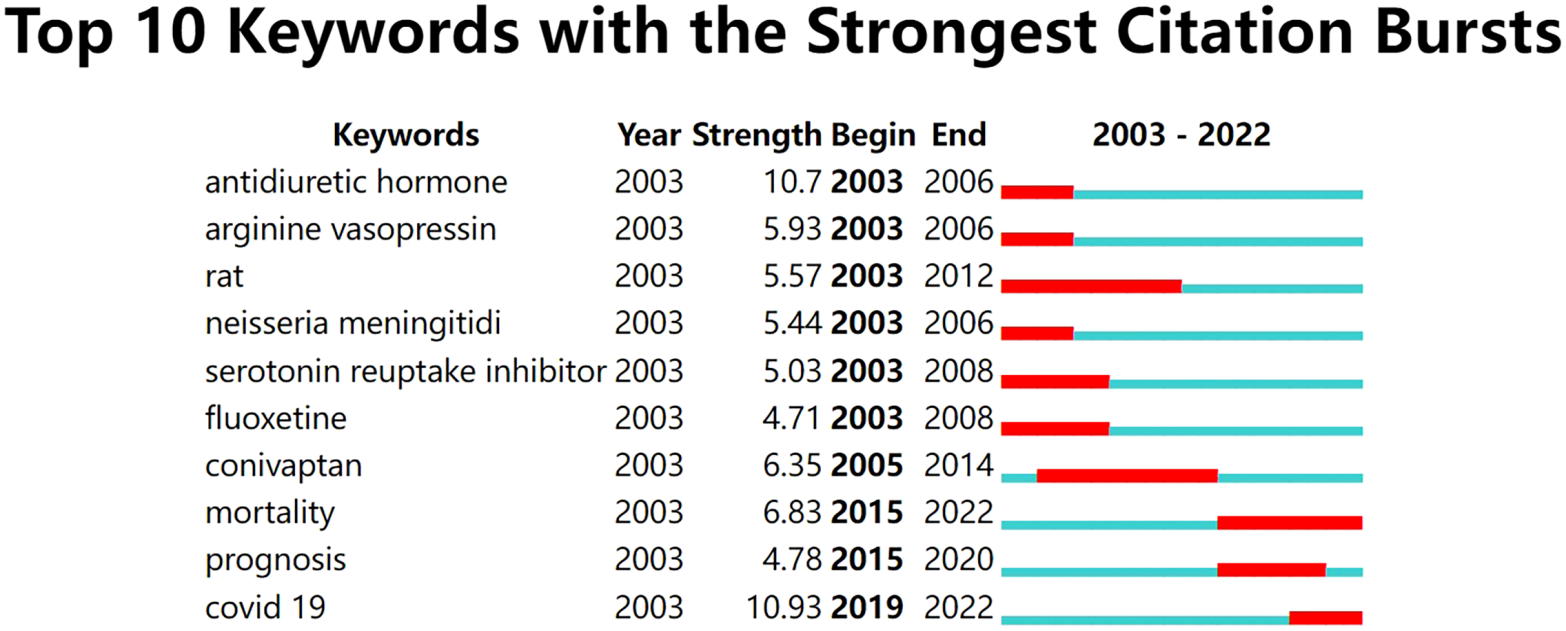
The top 10 Keywords with the strongest citation bursts in SIAD studies.

Keyword burst refers to keywords that appear frequently or have a high frequency of usage within a short period. Detection results usually include strength and temporal distribution, which not only can be beneficial to a retrospective review but also can predict phase-specific hotspots and research development trends in SIAD research. Keyword burst detection was implemented with CiteSpace (**Figure 8**). The blue lines represented the total time range, and the red lines represented the burst duration. Analysis showed that keywords, such as ‘antidiuretic hormone’, ‘arginine vasopressin’, ‘serotonin reuptake inhibitor’ and ‘fluoxetine’ appeared in the early years, but their burst periods only lasted 3–5 years. From 2003 to 2012, the keyword ‘rat’ received the largest attention, indicating that research focused on animal experiments. Recently, the most frequently used keywords were ‘mortality’ (2015–2022) and ‘covid-19’ (2019–2022), and ‘covid-19’ had the highest burst strength (10.93).

## 4 Discussion

A rising trend in the annual publication on SIAD was observed, indicating that relative research interest has attracted more attention worldwide in the last 20 years. Besides, this phenomenon may be attributed to increasing journals and researcher than before. The total publication and citation of the United States ranked first, reflecting that the United States’s increasing importance in SIAD research. Even though China was the third productive country, the relatively low average article citations (6.5) suggested that countries/institutions who have greater output but lower average article citations should pay more attention to further study and facilitate international cooperation in SIAD field.

The visualisation map based on countries, institutions and authors in the publication showed a close collaborative network among countries or regions, and the United States was the most prominent in international collaborations. Beaumont Hospital from the United States received the highest number of articles, and Georgetown University had the highest citation count with 1959 citations. Karolinska Institution and Karolinska University Hospital from a same group showed the tightest collaboration, which indicate that different institutions from same organization may have closer cooperation. Meanwhile, the cooperative relationship among other institutions appeared scattered, so research institutions around the world should further engage in frequent collaborations, which are conducive to SIAD research development and progress. Moreover, the frequency of citation manifests the impact of authors. Verbalis JG was the most co-cited author, whose work had been cited 520 times. This researcher contributed significantly to this field through multiple expert consensus and journal articles. In 2007, Ellison DH et al. published a clinical practice guideline in NEW ENGLAND JOURNAL MEDICINE, which was the highest co-cited literature. This guideline defined SIAD and systematically summarised the clinical manifestations, diagnosis, management and treatment of the syndrome (11).

Among the top 10 co-cited references, eight were related to hyponatremia. These articles introduced systematically the incidence, etiology, clinical manifestations, diagnosis, treatment and mortality related to hyponatremia. A clinical trial conducted by Schrier, R. W. et al. assessed the results of hyponatraemic patients treated with tolvaptan (15). A clinical research study found that mild chronic hyponatremia induced a high incidence of falls, which may contribute to marked gait and attention impairments (4). Besides, two articles within the top 10 most co-cited references focused on SIAD, including the first report and the evolution of clinical guidelines for SIAD (11, 16). Hyponatremia is the most common electrolyte disorder (3). The high citation rate of these articles showed that SIAD is closely related to hyponatremia and is the most common cause of hyponatremia (11).

There were 18 landmark articles in SIAD research identified by R version and co-citation analysis. The arginine vasopressin (AVP) activates the vasopressin 2 receptor in the collecting duct of the nephron where they mediate an antidiuretic response, and its inappropriate secretion result in SIAD. Leaf A et al. first reported study of the pathophysiological effects of vasopressin (pitressin) in humans in 1953 (19). This study revealed that pitressin hypersecretion leads to water retention and then increased urinary sodium excretion, so that it can explain why SIAD manifests as a pathogenesis of essentially normal fluid volume, hypotonic, hyposodium and increased urinary sodium. The measurement of arginine vasopressin levels in human plasma was first described by Robertson GL et al.in 1973 (20). It provided a basis for the precise measurement of AVP. Actually, the presence of AVP has the characteristics of short *in vitro* half-life and unstable, and even if stored under cryogenic conditions, it is still difficult to accurately measure, so it is still difficult to generalize in clinical practice. The first record on SIAD was published by Schwartz WB et al.in 1957 when two patients with bronchogenic carcinoma were diagnosed with SIAD (16). Bartter FC et al. published a review in The American Journal of Medicine in 1967. This influential review elucidated the pathophysiology mechanisms and clinical features of SIAD. It proposed the first diagnostic criteria, which greatly promoted the development of this field (13). Ellison DH et al. updated the diagnostic criteria for SIAD based on summary research into SIAD published before 2007; these criteria are still absolutely current (11). In SIAD, antidiuresis persists due to abnormal antidiuretic hormone secretion, which leads to impaired excretion of free water. According to the copeptin-based classification system of SIAD, SIAD can be subclassified into five patterns, known as types A to E. However, whether the treatment responses and prognosis assessment vary among these subtypes remains unclear (30). A prospective observational study revealed that the mortality rates of patients with SIAD is higher than those of normonatremic patients but lower than those of patients with hypovolemic and hypervolemic hyponatremia (27).

Considerable progress has been made in the management and treatment of SIAD. In 2006, Schrier RW et al. conducted two multicentre, randomised, double-blinded and placebo-controlled trials to evaluate the efficacy of tolvaptan, an oral vasopressin V2 receptor antagonist, in patients with euvolemic or hypervolemic hyponatremia. The study showed that tolvaptan effectively increased serum sodium concentrations at days 4 and 30 (15). Some studies suggested that tolvaptan alleviates hyponatremia and has few side effects (24). However, the European guidelines of hyponatremia published in 2014 considered increased risk for overly rapid correction of hyponatremia because of the use of vasopressin receptor antagonists (3). A multicentre study conducted by Tzoulis, P. et al. in 2016 confirmed that tolvaptan is associated with high risk of overcorrection and recommended that tolvaptan should be cautiously used with close electrolyte monitoring (25). At the same year, Verbalis, J. G. et al. published another essential clinical research in THE AMERICAN JOURNAL OF MEDICINE in 2016. This article reported that the most common monotherapy treatments for hyponatremia in SIAD include fluid restriction (48%), isotonic saline (27%), hypertonic saline (6%) and tolvaptan (13%); 11% of patients did not receive any active treatment, and all active treatments had greater mean rates in patients that showed serum sodium concentration change than the mean rates in patients without active treatments (26). Refardt J et al. explored the therapeutic effects of empagliflozin for the first time in an artificially induced SIAD model in 2017 (28). Subsequently, a randomised controlled trial evaluated the efficacy and safeness of empagliflozin for SIAD from 2016 to 2019; it reported that individuals with empagliflozin treatment had markedly higher plasma sodium levels than those who received placebos (31). Besides, no event of hypotension or hypoglycaemia was observed during the trial. Although 7% of patients treated with empagliflozin had a transient decrease in renal function, this potentially related adverse event was resolved (31). Additionally, a relatively new review exceeded our search range, which was published in 2023. This article shared a similar perspective that SGLT2i may benefit SIAD treatment (1). All these studies are closely related to studies on the mechanism of SGLT2i diuretics. A popular hypoglycaemic drug, SGLT2i inhibits the activity of SGLT2, decreases the renal glucose threshold and thereby increases urinary glucose excretion. These processes induce osmotic diuresis and increase water excretion. Thus, SGLT2i not only exerts a hypoglycaemic effect but also has a diuretic effect. Overall, SGLT2i may play a role in SIAD treatment.

Through keywords co-occurrence analysis, the top 10 keywords were closely related to the pathophysiological mechanism, differential diagnosis and treatment of SIAD. Keywords with high citation bursts are used or discussed frequently within a particular period. The most discussed topics related to SIAD in recent years were covid-19, prognosis and mortality, which seemed to be influenced by the coronavirus pandemic. The effects of covid-19 on SIAD, particularly those on therapeutic methods and prognostic evaluation of the syndrome, have become trending topics in SIAD research.

## 5 Limitations

This study objectively and systematically described bibliometric information with regard to articles about SIAD but still has some limitations. All data were extracted exclusively from WoSCC, which is the most frequently used database in bibliometric analysis. Moreover, the publication time of the literature influences citation rate. Thus, some significant articles may have been excluded. Extensive research is thus needed.

## 6 Conclusions

In the last 20 years, research related to SIAD has a steady growth. The United States plays a dominant role in this field. Beaumont Hospital was the most productive institution, while Georgetown University has the most citations in SIAD. Besides, cooperation among countries, institutions and authors should be strengthen. The influence of covid-19 on SIAD may be a core research topic. In conclusion, this bibliometrics analysis provided comprehensive insight into future SIAD research.

## Data availability statement

The datasets presented in this study can be found in online repositories. The names of the repository/repositories and accession number(s) can be found below: Web of Science Core Collection database.

## Ethics statement

This study is a bibliometric analysis of the articles related to Syndrome of inappropriate antidiuretic hormone secretion, and the data were obtained from the Clarivate Analytics Web of Science Core Collection database. Thus, this study did not involve intervention or data collection in animal experiments or clinical trials, so ethical approval and consent to participate were not necessary for this paper.

## Author contributions

XW: Writing – original draft, Data curation, Formal analysis, Methodology, Resources, Visualization. WP: Writing – original draft, Data curation, Formal analysis, Methodology, Resources, Visualization. SL: Writing – review & editing, Methodology. YW: Writing – review & editing, Data curation. YC: Writing – review & editing, Resources. TF: Writing – review & editing, Resources. XC: Writing – review & editing, Project administration, Supervision. ML: Project administration, Supervision, Writing – review & editing.
